# Joint Hypermobility and Clinical Correlates in a Group of Patients With Eating Disorders

**DOI:** 10.3389/fpsyt.2021.803614

**Published:** 2022-01-12

**Authors:** Carolina Baeza-Velasco, Maude Seneque, Philippe Courtet, Émilie Olié, Charles Chatenet, Paola Espinoza, Géraldine Dorard, Sébastien Guillaume

**Affiliations:** ^1^Université de Paris, Laboratoire de Psychopathologie et Processus de Santé, Boulogne Billancourt, France; ^2^Department of Emergency Psychiatry and Acute Care, CHU Montpellier, Montpellier, France; ^3^Institute of Functional Genomics, University of Montpellier, CNRS, INSERM, Montpellier, France; ^4^Departament de Psicologia, Unitat d'Avaluació i Intervenció en Imatge Corporal, Clínica i de la Salut, Universitat Autònoma de Barcelona, Barcelona, Spain

**Keywords:** eating disorders, joint hypermobility, anorexia nervosa, cognitive rigidity, pain

## Abstract

**Background:** The ability to move joints beyond the normal range of motion is called Joint Hypermobility (JHM). JHM has been associated with a plethora of physical problems and is a frequent sign of hereditary disorders of connective tissue. Neuropsychiatric conditions such as eating disorders (ED) have also been related to JHM. However, little is known about the clinical profile of people with ED and JHM. The aim of this study was to explore JHM in patients with ED and to compare the clinical characteristics of hypermobile ED patients with non-hypermobile ED patients.

**Method:** Fifty-three outpatients diagnosed with ED were assessed using the Beighton score for JHM, the Eating Disorders Inventory 2, the Eating Disorder Examination, and the Detail and Flexibility Questionnaire. Information relating to patients' psychiatric and somatic comorbidities/symptoms was also collected.

**Results:** Using the traditional Beighton score's cutoff of ≥4, 41.5% of the sample presented with JHM. Our results indicate that compared with non-hypermobile ED patients, those with JHM are significantly younger, suffer at a greater extent from joint pain and easy bruising, have a shorter duration of the ED, and have lower scores for cognitive rigidity. In addition, for those with anorexia nervosa, the restricting subtype represents a significantly lower proportion of hypermobile ED patients compared to non-hypermobile ED patients. Multivariate analyses showed that cognitive rigidity, age, and duration of the ED could predict the JHM status in this sample.

**Conclusion:** These results suggest that JHM is frequent in patients with ED and is accompanied by signs of tissue fragility. Patients with ED and JHM seem to present a specific profile characterized by less cognitive rigidity and restricting behavior in the case of anorexia nervosa. Further research is needed in order to confirm these results.

## Introduction

Joint hypermobility (JHM) is a somatic trait characterized by an increased range of joint motion. According to studies of general populations, the prevalence of JHM ranges from 10 to 20% (1, 2). However, JHM is more frequent in females and in young people since JHM declines with age (3). When JHM is generalized to many joints, it is supposed to be congenital, inherited, and related to connective tissue (4). Thus, the presence of JHM often underlies abnormal collagen production. In such a context, it is not surprising that JHM has been related to several physical problems of both articular and non-articular nature, with varying severity. The most severe cases are the so-called hereditary disorders of connective tissue (e.g., Ehlers-Danlos syndromes, Marfan syndrome, and osteogenesis imperfecta), of which JHM is a hallmark.

JHM has been also related to neuropsychiatric conditions, especially anxiety disorders [e.g., (5)], neurodevelopmental disorders such as autism [e.g., (6)], and attention-deficit/hyperactivity disorder [e.g., (7)]. Although rare, some works highlight a link between JHM and hypermobility-related disorders and/or eating disorders (ED). To the best of our knowledge, Goh et al. (8) were the first to report results from a systematic study, wherein JHM was assessed in 30 patients with anorexia nervosa (AN), their first-degree relatives (*n* = 29), and heathy controls (*n* = 16). Results showed that JHM was more frequent in patients than in relatives and controls (63, 34, and 13%, respectively). The authors concluded that “JHM is a possible indicator of a familial disorder of connective tissue which potentially plays a causal role in the development of ED” (8). In another study, Bulbena-Cabr et al. (9) reported that hypermobile non-clinical youngsters scored significantly higher than non-hypermobile individuals on bulimia and anorexia subscales of the Spanish version of the Body Perception Questionnaire (10). Later, the same team explored JHM in a group of children affected by ARFID (avoidant/restrictive food intake disorder). They observed that compared to healthy controls, a significantly major proportion of these children presented with JHM (11). Recently, our group reported that patients with Ehlers-Danlos syndrome (mainly affected by the hypermobile subtype) showed an increased prevalence of ED history, higher risk of current ED, and lower BMI than healthy controls (12).

Although preliminary, these data indicate the potential relationship between collagen conditions and ED. In this sense, it is possible that gastrointestinal problems that are frequent in hypermobility conditions (13)—as well as food allergies, fragility of oral mucosa, chemo-sensorial abnormalities, and proprioception problems which may impact the body schema and image—constitute a favorable environment for the development of ED (14). Thus, more studies are needed in order to confirm previous results and elucidate the clinical profile of people presenting with such a mixed picture of conditions.

The aim of this study was to explore JHM in patients with ED and to compare the clinical characteristics of hypermobile ED patients with non-hypermobile ED patients.

## Methods

### Participants

In this study, the participants were men and women clinically diagnosed with ED at the outpatient ED unit of the University Hospital of Montpellier (France). In this unit, diagnoses are established by a multidisciplinary team composed of psychiatrists, psychologists, and nutritionists. Through this process, a consensus is reached using the best estimated procedure, medical records and information from relatives, non-standardized clinical assessments by practitioners, and standardized measures.

Patients with different types of ED were enrolled between June 2019 and March 2021. Exclusion criteria included pregnancy, not being fluent in French, and refusal to participate in the study.

### Instruments

#### The Beighton Score for JHM

The Beighton score (15) is the most widely used and probably the most reliable tool to assess JHM (4). It consists of the execution of five maneuvers with a maximum score of nine points: (1) passive dorsiflexion of the fifth metacarpal joint (one point per side), (2) passive thumb opposition to the forearm (one point per side), (3) passive hyperextension of the elbow (one point per side), (4) passive hyperextension of the knee (one point per side), (5) the capacity to place both hands flat on the floor in front of the feet with the knees straight (one point). The traditional cutoff score of ≥4 out of nine points recommended by Beighton's original work was used in this study to identify JHM.

#### The Mini International Neuropsychiatric Interview (MINI 5.0.0)

The MINI (16) is a structured interview which enables the positive diagnosis of mental disorders through questions phrased to allow yes/no answers. This tool was used to assess psychiatric comorbidities (lifetime anxiety disorders, major depressive disorder, and suicide attempts are reported in this study). Version 5.0.0 of the MINI is based on the Diagnostic and Statistical Manual of Mental Disorders, fourth edition [DSM-IV; (17)] and the International Classification of Diseases, 10th edition—psychiatric disorders [ICD-10; (37)].

#### The Eating Disorders Inventory 2 (EDI-2)

The EDI-2 (18, 19) is a self-report diagnostic tool designed to evaluate symptoms of ED as well as its relation to personality traits and emotions through 91 items rated on a six-point Likert scale. It contains 11 subscales: drive for thinness, bulimia, body dissatisfaction, ineffectiveness, perfectionism, interpersonal distrust, interoceptive awareness, maturity fears, asceticism, impulse regulation, and social insecurity. The higher the score, the greater the ED symptoms. In the present study, Cronbach's α for the total scale was 0.92.

#### The Eating Disorder Examination (EDE-Q)

The self-questionnaire EDE-Q (20) is a screening tool that assesses, through 28 items, the four core clinical dimensions of ED: eating concern, body shape concern, weight concern, and restraint. The total score is calculated by adding all subscales together and then dividing the result by four. Higher scores indicate a more severe ED symptomatology. Cronbach's α for the global score was 0.76.

#### The Detail and Flexibility Questionnaire (DFlex)

The DFlex (21) consists of a 24-item self-report scale assessing cognitive rigidity (difficulty with set-shifting/flexibility) and over-attention to detail (weak coherence). These cognitive styles are typical of rigid perfectionism in patients with ED. Each item of the DFlex is presented using a rating Likert scale from 1 (“strongly disagree”) to 6 (“strongly agree”). Higher scores indicate higher levels of psychopathology. Cronbach's α for the total scale was 0.88.

### Fatigue and Pain

Perceived levels of fatigue in the final week of the study were obtained by participants scoring their general level of fatigue in that week on a numeric scale ranging from 0 to 10. Scores ≥ 6 were categorized as moderate/severe fatigue.

Additional clinical/health information was obtained from the clinical interview (BMI, somatic comorbidity, fractures, and age of menarche) and through an *ad hoc* self-questionnaire with yes/no questions about somatic problems frequently observed in hypermobile subjects (i.e., easy bruising, joint pain, dislocations, thin skin, varicose veins, stretch marks, and physiotherapy consultation).

### Statistical Analysis

Statistical treatment included a descriptive analysis of the data, non-parametric tests (Chi-square and Spearman correlation), and binary logistic regression. Data analysis was performed with the IBM SPSS version 28 software package, and the significance level was taken as 0.05 for all statistical tests.

## Results

Fifty-three patients with ED were included in this study (AN = 67.9%; bulimia nervosa = 18.8%; other ED = 13.2%) Most of them were female (92.5%). The sample mean age was 27.6 (SD = 10.4) (Table 1).

**Table 1:** Comparison of characteristics between ED patients with and without JHM.

**Variable**	**ED without JHM 58.5% [31]**	**ED with JHM 41.5% [22]**	**OR [95% CI]**	***P*-value**
**Sociodemographics**	**% [*n*]/Mean (SD)**	**% [*n*]/Mean (SD)**		
Age	31.03 (11.02)	22.77 (2.07)	0.904 [0.839; 974]	0.008
Women	87.1% [27]	100% [22]	-	0.080^*^
Years of study	13.68 (2.40)	12.90 (2.07)	0.854 [0.656; 1.113]	0.244
**ED characteristics**				
Age at onset	17.60 (5.63)	16.35 (3.16)	0.939 [0.816; 1.080]	0.379
Age first consultation	23.77 (9.53)	18.00 (2.64)	0.871 [0.752;1.010]	0.067
Duration of ED (in years)	12.93 (9.07)	6.25 (6.73)	0.891 [0.811; 0.978]	0.015
Anorexia nervosa	61.2% [19]	77.2% [17]	2.147 [0.627; 7.358]	0.224
Type of anorexia nervosa:			4.66 [1.108; 19.652]	0.036
Restricting type	73.7% [14]	37.5% [6]		
Bingeing/purging type	26.3% [5]	62.5% [10]		
Bulimia nervosa	19.3% [6]	18.1% [4]	0.980 [0.240; 4.004]	0.978
Other ED	19.3% [6]	4.5% [1]	0.219 [0.024; 1.978]	0.176
**Psychiatric comorbidity (lifetime)**				
Major depressive disorder	75.9% [22]	62.5% [10]	0.530 [0.141;1.98]	0.347
Suicide attempt	30% [9]	14.3% [3]	0.389 [0.091; 1.659]	0.202
Anxiety disorders	58.6% [17]	66.7% [10]	1.412 [0.383; 5.19]	0.604
Substance abuse and/or dependence	10.3% [3]	13.3% [2]	1.33 [0.198; 8.996]	0.768
**Somatic/health variables (lifetime)**				
BMI	19.31 (6.2)	20.22 (4.8)	1.029 [0.924; 1.134]	0.564
Age of menarche	13.76 (2.43)	12.67 (.976)	0.689 [0.422; 1.125]	0.136
Somatic comorbidity	25.8% [8]	31.8% [7]	1.342 [0.402; 4.477]	0.633
Fractures	19.4% [6]	4.5% [1]	0.198 [0.022; 1.782]	0.149
Dislocations	10.7% [3]	9.5% [2]	0.877 [0.133; 5.783]	0.892
Easy bruising	57.1% [16]	95.2% [20]	15.0 [1.759; 127.913]	0.013
Moderate and severe fatigue (usual)	80.6% [25]	77.3% [17]	0.816 [0.214; 3.108]	0.766
Moderate and severe pain (usual)	35.5% [11]	61.9 [13]	2.955 [0.938; 9.309]	0.064
Joint pain	25% [7]	57.1% [12]	4.00 [1.186; 13.495]	0.025
Thin skin	17.9% [5]	42.9% [9]	3.45 [0.943; 12.621]	0.061
Varicose veins	14.3% [4]	4.8% [1]	0.300 [0.031; 2.904]	0.299
Stretch marks	50% [14]	61.9% [13]	1.625 [0.514; 5.136]	0.408
Physiotherapy consultation	35.7% [10]	61.9% [13]	2.925 [0.906; 9.442]	0.073
**Psychological dimensions**				
**Detail and flexibility questionnaire:**				
Cognitive rigidity	51.43 (8.16)	45.15 (12.14)	0.939 [0.884; 998]	0.041
Attention to detail	43.60 (8.66)	39.65 (12.15)	0.962 [907; 1.019]	0.186
**Eating disorders examination questionnaire:**				
Restraint	3.50 (2.55)	2.20 (2.16)	0.787 [0.490; 1.265]	0.323
Eating concern	2.43 (1.90)	1.00 (1.41)	0.539 [0.148; 1.958]	0.347
Weight concern	3.20 (1.64)	4.25 (.957)	1.904 [0.621; 5.837]	0.260
Shape concern	4.80 (1.64)	5.25 (.957)	1.366 [0.426; 4.379]	0.600
**Eating disorders inventory 2:**				
Drive for thinness	4.46 (3.89)	4.45 (4.74)	0.999 [0.872; 1.145]	0.989
Bulimia	5.43 (4.15)	5.80 (4.37)	1.021 [0.892; 1.170]	0.760
Body dissatisfaction	4.76 (2.97)	3.80 (2.87)	0.885 [7.17; 1.094]	0.259
Ineffectiveness	6.56 (3.74)	7.10 (4.37)	1.035 [0.896; 1.195]	0.639
Perfectionnisme	4.73 (3.59)	6.00 (3.12)	1.118 [0.942; 1.328]	0.202
Interpersonal distrust	4.63 (2.69)	4.25 (2.98)	0.951 [0.772; 1.170]	0.632
Interoceptive awareness	8.66 (4.48)	8.55 (4.75)	0.994 [0.877;1.127]	0.928
Asceticism	7.26 (2.65)	7.05 (2.34)	0.966 [767; 1.216]	0.769
Impulse regulation	12.76 (5.83)	12.15 (5.46)	0.981 [0.885; 1.087]	0.711
Social insecurity	4.60 (2.13)	5.10 (2.53)	1.095 [0.857; 1.400]	0.468
Maturity fears	6.62 (3.02)	6.00 (3.75)	0.943 [0.789; 1.128]	0.522

*ED, eating disorder; BMI, body mass index; CI, Confidence Interval; OR, Odds ratio; SD, standard deviation;*

* *, chi-square*.

Twenty-two patients (41.5%) scored ≥ 4 for the Beighton score for JHM (i.e., the hypermobile group). When hypermobile and non-hypermobile ED patients were compared using sociodemographic data, we observed that those with JHM were significantly younger (22.7 vs. 31; *p* = 0.008).

Concerning clinical characteristics (Table 1), the non-hypermobile group had a longer duration of the ED in terms of years (12.9 vs. 6.2; *p* = 0.015), and those with AN presented with the restricting subtype to a greater extent compared to hypermobile ED patients (73.7 vs. 26.3%; *p* = 0.014). In addition, the non-hypermobile ED patients had higher scores for cognitive rigidity than hypermobile individuals (51.4 vs. 45.1; *p* = 0.041). No other difference was found between the groups on measures of psychological functioning.

With respect to somatic aspects, a significantly greater proportion of the hypermobile ED patients presented with easy bruising (95.2 vs. 57.1%; *p* = 0.013) and joint pain (57.1 vs. 25%; *p* = 0.025) compared to the non-hypermobile group. The results are summarized in Table 1.

Binary logistic regression using JHM status as a dependent variable and duration of ED (highly correlated with age), AN subtype, cognitive rigidity, and joint pain as covariates showed that cognitive rigidity and duration of ED can distinguish between hypermobile and non-hypermobile ED patients.

A second model using age, AN subtype, cognitive rigidity, and joint pain as covariates showed that the age and cognitive rigidity could predict JHM status (Table 2).

**Table 2:** Multivariate regression logistic models with JHM status as dependent variable.

**Model 1**	**OR [95%CI]**	***P-*value**
Duration of ED	0.847 [0.719; 997]	0.047
Anorexia nervosa subtype	7.769 [0.798; 75.634]	0.077
Cognitive rigidity	0.878 [0.777; 0.992]	0.037
Joint pain	0.880 [0.102; 7.62]	0.908
**Model 2**	**OR [95%CI]**	***P*** **-value**
Age	0.850 [0.730; 0.990]	0.036
Anorexia nervosa subtype	9.567 [0.894; 102.43]	0.062
Cognitive rigidity	0.872 [0.779; 0.975]	0.017
Joint pain	0.723 [0.081; 6.485]	0.772

*CI, Confidence Interval; OR, Odds ratios; ED, eating disorders*.

## Discussion

The aim of the present study was to explore JHM in patients with ED as well as their clinical characteristics. As expected, a high proportion of patients with ED (41.5%) were positive for JHM using the Beighton score, which is greater than the prevalence reported for the general population [10–20%; (1, 2)]. However, we observed a lower frequency of JHM in those with AN than that observed by the only study allowing comparisons. Indeed, Goh et al. (8) reported that 63% of their sample composed of 30 AN patients presented with JHM, while in our sample, 47.2% of AN patients (*n* = 36) presented with JHM. A possible explanation for this difference is the method used to assess JHM, which is not specified in the report of Goh et al. In any case, these results confirm previous reports suggesting an over-representation of JHM in people affected by ED (9, 11, 12, 14). However, it is possible that the link between JHM and ED varies depending on the ED type. Accordingly, in the present study, no relationship was found between bulimia nervosa (another type of ED) and JHM. In contrast, although from a purely descriptive point of view, AN appears to be more frequent among hypermobile patients than among non-hypermobile patients (77.2 vs. 61.2%). In addition, we observed significant differences between hypermobile and non-hypermobile AN patients with respect to AN subtype. The non-hypermobile group mostly presented with the restricting subtype, while the hypermobile group mostly presented with the binging/purging subtype. Interestingly, this result is in line with the fact that non-hypermobile patients scored significantly higher for cognitive rigidity. Indeed, there is evidence that these two AN subtypes differ with respect to cognitive style (22), with the restricting subtype corresponding to more cognitive control (23). Plausibly, the younger age of the hypermobile ED group (22 vs. 31 y/o) could have influenced the results for cognitive rigidity, since several studies have reported that young patients with AN are unaffected by processes compromised by cognitive rigidity (e.g., set-shifting). This suggests that these cognitive impairments increase as a consequence of illness (24). However, from multivariate analysis, cognitive rigidity appears to be a predictor even when age and duration of ED are controlled. Thus, these results suggest that hypermobile ED patients may present a specific profile characterized by less cognitive rigidity and restricting behavior. A clue for understanding this finding comes from the results of the neuroimaging study by Eccles et al. (25), who found structural brain differences between hypermobile and non-hypermobile subjects—for instance, in the dorsal right anterior cingulate cortex, which is an area engaged in cognitive control (26, 27).

We observed that the hypermobile ED group was significantly younger than the non-hypermobile group (22 vs. 31 y/o), which is not surprising. As mentioned before, JHM is more common in children and decreases as age increases (3). Accordingly, the duration of ED in terms of years was also lower in the hypermobile ED group.

Interestingly, while the age of ED onset was similar in both groups, patients from the hypermobile ED group had their first ED consultation during adolescence and at a younger age than non-hypermobile patients; however, this difference did not reach statistical significance (18 vs. 23 y/o; *p* = 0.067). Since congenital JHM is frequently accompanied by several physical problems, it is possible that hypermobile ED patients are more familiar with medical consultation from an early age. In this sense, our data show that 61% of patients with JHM consulted the physiotherapist vs. 35% of non-hypermobile patients. In addition, symptoms derived from or related to collagen-altering conditions are often exacerbated with puberty, probably due to estrogen secretion and the fast growth of tissue (e.g., skin, joints, and muscles) during this period (28). Thus, this context constitutes a fertile ground for the development of mental pathologies such as ED. Indeed, among the plethora of physical problems experienced by people with hypermobility-related disorders, there are many that affect food intake (14). For instance, functional gastrointestinal problems [e.g., abdominal pain, vomiting, dysphagia, bloating, gastro-esophageal reflux; (13)], teeth and temporomandibular problems (29), food allergies (30), oral mucosa fragility (31), and chemosensory particularities [e.g., hyperosmia; (32)] may perturb eating, thereby promoting disordered eating behavior (14).

On the other hand, tissue fragility and proprioception impairment, which are common in people with JHM (33), may affect the development of a secure sense of body and an accurate body image, which are core factors of ED (34). Thus, there are reasonable grounds for considering hypermobile subjects as a group particularly vulnerable to ED. According to our results, we can speculate that in the context of JHM, ED is probably secondary to the connective tissue problems rather than attributable to the premorbid perfectionism and rigid cognitive style classically described in AN, and that the presenting symptomatology of these patients is somewhat mixed rather than strictly restrictive.

Unsurprisingly, a significantly greater proportion of the hypermobile group suffered from easy bruising and joint pain compared to the non-hypermobile group. These symptoms are strongly associated with JHM (35) and reflect the tissue fragility underlying collagen defects (e.g., fragility of capillaries and blood vessels surrounding the connective tissue, weakness of ligaments holding the bones of joints, and reduced joint stability). From a clinical perspective, these signs and symptoms are of particular interest and should not be overlooked. Indeed, the significant weight loss and consequent poor nutrition that occur in ED can further weaken patients who already have an inherently fragile body. For instance, Kaplan and Katz (34) stated that amenorrhea secondary to weight loss may affect bone physiology. Thus, in patients with a connective tissue disorder (of which JHM is a hallmark) long-term amenorrhea and bone mass loss may lead to serious fractures. In addition, in more severe cases of heritable disorders of connective tissue (e.g., Ehlers-Danlos syndromes), there may be a risk of visceral perforation in patients with significant weight loss combined with intense physical exercise (36).

The present study has important limitations, such as the use of a small sample of convenience, which compromises the generalization of results. In addition, the cross-sectional design does not allow any conclusions about the directionality of the relationships between the different variables. Despite these limitations, our results add weight to the scarce but growing evidence about the relationship between ED and JHM. Further studies are needed to confirm these findings and illustrate a clearer picture of the co-occurrence between these two conditions.

## Data Availability Statement

The data that support the findings of this study are available from the corresponding author (CB-V), upon reasonable request.

## Ethics Statement

The studies involving human participants were reviewed and approved by CPP Sud Méditerranée IV. Written informed consent to participate in this study was provided by the participants' legal guardian/next of kin.

## Author Contributions

CB-V and SG designed the study. SG, MS, and CC assisted with the assessment of patients and data collection. CB-V performed the statistical analysis and drafted the manuscript. All authors have reviewed the manuscript for intellectual content and have approved the final manuscript.

## Conflict of Interest

The authors declare that the research was conducted in the absence of any commercial or financial relationships that could be construed as a potential conflict of interest.

## Publisher's Note

All claims expressed in this article are solely those of the authors and do not necessarily represent those of their affiliated organizations, or those of the publisher, the editors and the reviewers. Any product that may be evaluated in this article, or claim that may be made by its manufacturer, is not guaranteed or endorsed by the publisher.
